# Failure of non-invasive respiratory support after 6 hours from initiation is associated with ICU mortality

**DOI:** 10.1371/journal.pone.0251030

**Published:** 2021-04-30

**Authors:** Mitsuaki Nishikimi, Kazuki Nishida, Yuichiro Shindo, Muhammad Shoaib, Daisuke Kasugai, Yuma Yasuda, Michiko Higashi, Atsushi Numaguchi, Takanori Yamamoto, Shigeyuki Matsui, Naoyuki Matsuda

**Affiliations:** 1 Department of Emergency and Critical Care Medicine, Nagoya University Graduate School of Medicine, Nagoya, Japan; 2 Department of Emergency Medicine, Northwell Health, NY, NY, United States of America; 3 Laboratory for Critical Care Physiology at the Feinstein Institutes for Medical Research, Northwell Health, NY, NY, United States of America; 4 Department of Biostatistics, Nagoya University Graduate School of Medicine, Nagoya, Japan; 5 Department of Respiratory Medicine, Nagoya University Graduate School of Medicine, Nagoya, Japan; 6 Donald and Barbara Zucker School of Medicine at Hofstra/Northwell, Hempstead, NY, United States of America; Kaohsuing Medical University Hospital, TAIWAN

## Abstract

A previous study has shown that late failure (> 48 hours) of high-flow nasal cannula (HFNC) was associated with intensive care unit (ICU) mortality. The aim of this study was to investigate whether failure of non-invasive respiratory support, including HFNC and non-invasive positive pressure ventilation (NPPV), was also associated with the risk of mortality even if it occurs in the earlier phase. We retrospectively analyzed 59 intubated patients for acute respiratory failure due to lung diseases between April 2014 and June 2018. We divided the patients into 2 groups according to the time from starting non-invasive ventilatory support until their intubation: ≤ 6 hours failure and > 6 hours failure group. We evaluated the differences in the ICU mortality between these two groups. The multivariate logistic regression analysis showed the highest mortality in the > 6 hours failure group as compared to the ≤ 6 hours failure group, with a statistically significant difference (*p* < 0.01). It was also associated with a statistically significant increased 30-day mortality and decreased ventilator weaning rate. The ICU mortality in patients with acute respiratory failure caused by lung diseases was increased if the time until failure of HFNC and NPPV was more than 6 hours.

## Introduction

Many previous studies have shown the usefulness of non-invasive respiratory support, including high-flow nasal cannula (HFNC) and non-invasive positive pressure ventilation (NPPV), for patients with acute respiratory failure in the intensive care unit (ICU) [1–3]. It is suggested that the outcomes of patients with pulmonary diseases admitted to the ICU could be improved by the application of these measures [4–6], although the precise underlying mechanisms remain controversial [7]. If a trial of such non-invasive respiratory supports succeeds, an improvement in the degree of oxygen saturation that cannot be obtained with conventional oxygen supplementation may be expected, diminishing and/or precluding the need for invasive intubation of the patient [8–10]. However, if such a trial fails and the patients eventually need to be intubated, then the mortality in such patients may be worse as compared to that in patients who have undergone primary intubation [11–14].

Recently, Kan BJ et al. divided 175 patients with failure of HFNC into two groups, according to whether the intubation was performed early (within 48 hours; early failure group) or late (at least 48 hours; late failure group); the late failure group showed increased ICU mortality, as well as decreased extubation success rate, ventilator-free days, and ventilator weaning rate as compared to the early failure group [15]. They concluded that 48 hours for failure of HFNC might result in delayed intubation and worse clinical outcomes in patients with respiratory failure. However, it remains unclear whether the 48 hour window was the most appropriate cutoff point for to determine the failure of non-invasive respiratory support. Given that a positive response to treatment should certainly be evident at 4–6 hours after start of the support [16, 17] and the cutoff points of 6 hours is more common in actual clinical practice to judge the effect of the support rather than 48 hours [18–20], there is a worthwhile evaluating whether the mortality of patients with failure of HFNC/NPPV could be increased even if the patients with failure are intubated earlier than 48 hours, namely 6 hours, after the start of the non-invasive support.

Therefore, in the present study, we investigated whether even earlier failure of HFNC/NPPV would be associated with increased mortality. We divided all intubated patients into 2 groups (1: ≤ 6 hours from the initiation of non-invasive respiratory support to intubation [≤ 6 hours failure group]; 2: > 6 hours from the initiation of non-invasive support to intubation [> 6 hours failure group]) and evaluated the ICU mortality in these two groups.

## Materials and methods

### Study design

We conducted this single-center, retrospective, observational study in adult patients admitted to our ICU between April 2014 and June 2018. Nagoya University Hospital is an academic and educational hospital with 1,035 beds, including 10 emergency and medical ICU (EMICU) beds and 16 surgical ICU beds. This study was performed in the EMICU, and the subjects were patients admitted via the emergency department (emergency ICU), or medical patients admitted via the general ward (medical ICU) who needed critical care treatment. All patients in EMICU were treated by dedicated intensivists and nurses who are assigned as fulltime ICU staff. The eligibility criteria were intubated adult patients (≥ 18 years old) with acute respiratory failure due to lung disease. Patients were excluded if they were already tracheotomized prior to the ICU admission. Patients were also excluded if they were intubated or underwent non-invasive respiratory support in other departments or other hospitals, because the data prior to ICU admission could not be obtained. Patients were also excluded if they were intubated without a trial of non-invasive respiratory support. As such, we assessed the patients who were underwent non-invasive respiratory support at our EMICU and emergency department. In this study, we divided all intubated patients into 2 groups; 1: ≤ 6 hours failure group (patients who were intubated after an attempt of non-invasive respiratory support for less than 6 hours); 2: > 6 hours failure group (patients who were intubated after an attempt of non-invasive respiratory support for more than 6 hours). The cutoff time point of 6 hours was clinically decided because some guidelines recommend 6 hours as one of the most appropriate time windows to evaluate the effect of non-invasive respiratory support [16, 21]. This study was conducted with the approval of the research ethics boards of Nagoya University Hospital, which waived the requirement for obtaining of informed patient consent from the study participants to ensure participant anonymity, as stipulated in the Japanese government guidelines.

### Data set

Data was collected retrospectively from the patients’ electronic health records. Data includes the clinical history/patient characteristics (age, sex, past illnesses, etc.), vital signs (blood pressure, heart rate, percutaneous oxygen saturation (S_p_O_2_), etc.), results of blood gas and chemistry analysis, and the clinical course after patient admission. The Sequential Organ Failure Assessment (SOFA) score was calculated using the worst values of the relevant variables within the first 24 hours after ICU admission. The underlying pulmonary diseases causing the respiratory failure were classified into 5 groups according to the assessment of the attending ICU physicians and pulmonologists during patient admission: infectious lung disease, interstitial lung disease, obstructive airway disease, including chronic obstructive pulmonary disease (COPD) and asthma, extrapulmonary acute respiratory distress syndrome (ARDS), and others (unknown etiology of ARDS, 11 patients; pulmonary embolism, 1 patient; alveolar hemorrhage, 6 patients; traumatic lung injury, 3 patients). The time of admission to the ICU, time of initiation of HFNC/NPPV, intubation, and extubation were obtained from nursing records. We defined the time until failure of the non-invasive respiratory support as the time from the start of the support (HFNC/NPPV) until intubation.

### Outcome

The primary outcome was the mortality rate at discharge from the ICU. For secondary outcomes, we evaluated 30-day mortality and the successful ventilator weaning rates at discharge from the ICU. Ventilator weaning rate at discharge refers to the percentage of patients who were successfully weaned off mechanical ventilatory support by the time of discharge from the ICU.

### Application of high-flow nasal cannula and non-invasive ventilation

We followed the established guideline for the application of NFNC/NPPV and the judgement of failure [21]. We used an HFNC (Optiflow, Fisher and Paykel Healthcare, Auckland, New Zealand) or NPPV (V60 ventilator, Philips Healthcare, Amsterdam, Nederland) for eligible patients, namely, patients with hypoxia who required oxygen supplementation at the rate of ≥10 L/min via a conventional oxygen mask device to achieve an SpO_2_ of >92%, and those who showed persistent signs of respiratory distress, namely, a respiratory rate of >24/min and use of accessory respiratory muscles despite adequate oxygen supplementation. We considered primary intubation without a trial of HFNC/NPPV for patients with severe hypoxia (PaO2/FIO2 < 100 mm Hg), respiratory acidosis (pH < 7.3), excessive sputum and/or severe lung atelectasis. In other cases, preference was given to HFNC/NPPV over tracheal intubation. If the goal was to improve the oxygen saturation as well as reduce carbon dioxide accumulation, we tended to select NPPV. On the other hand, if the patient was very restless, we tended to select HFNC rather than NPPV.

Under these circumstances, the ICU doctors examined the patients’ respiratory status every 30 minutes by examining vital signs, breathing pattern, results of blood gas examination (if needed), etc. They decided to intubate the patient as soon as possible after they determine no beneficial effect of the use of HFNC or NPPV. We judged failure of HFNC/NPPV under the following circumstances: persistent hypoxia (SpO_2_ <92%), hypercapnia with acidosis (pH <7.3), and signs of respiratory distress (respiratory rate >24/min and use of accessory respiratory muscles), together with circulatory failure or coma despite maximum HFNC/NPPV support (HFNC: 100% FiO2 and 60 L/min; NPPV: 100% FiO2 and PEEP 15 cm H_2_O). When required, we also attempted to change the device used for non-invasive ventilation prior to intubation (from HFNC or NPPV to another device). In regard to the intubation, we performed rapid-sequence intubation for all patients using fentanyl, midazolam, and rocuronium.

### Statistics

In the analysis of baseline characteristics, Fisher’s exact test was used to compare the categorical data, and Mann-Whitney U test for continuous variables. Multivariate analysis was performed with adjustments for 3 factors (model 1; age, SOFA score and P_a_CO_2_ before intubation) or 5 factors (model 2; age, reason for acute respiratory failure, SOFA score, P_a_O_2_/FiO_2_ ratio before intubation, and P_a_CO_2_ before intubation) that are clinically considered as potentially exerting significant influence on outcomes [22–24]. To investigate and visualize non-linear relationships of time to failure that affect ICU mortality, we applied a logarithmic transformation and used a generalized additive model (GAM) with splines, using the same 3 adjustment factors as mentioned above. Survival analysis was performed using the Kaplan-Meier method. Survival time was defined as the interval between intubation and death, or the 30-day survival rate from intubation. Difference between the ≤ 6 hours failure group and > 6 hours failure group was tested by the log-rank test. All reported p values were two-sided, and *p* < 0.05 was regarded as denoting a statistically significant difference. All analyses were conducted using R, version 3.5.1.

## Results

### Patient flow and baseline characteristics

**Fig 1:**
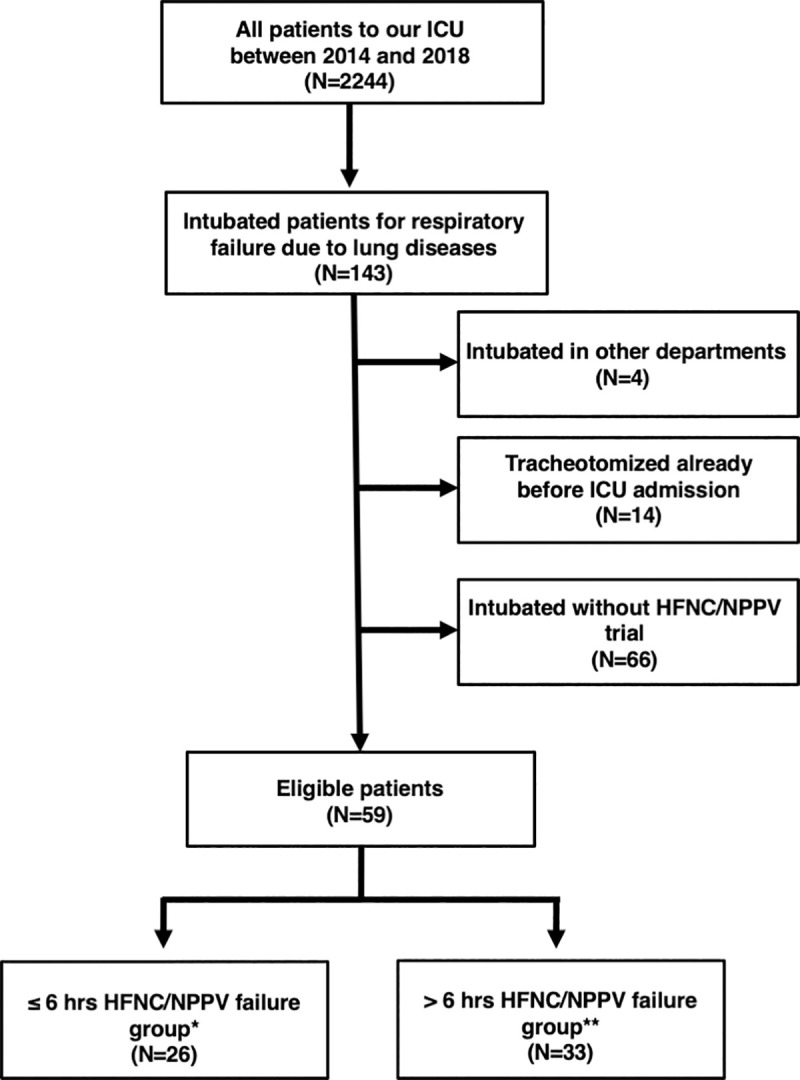
Patients flow. ICU = intensive care unit; HFNC = high-flow nasal cannula; NPPV = noninvasive positive pressure ventilation. * group showing failure of HFNC/NPPV ≤ 6 hours of its initiation. ** group showing failure of HFNC/NPPV > 6 hours after its initiation.

Among the 2,244 patients admitted to our ICU between April 2014 and June 2018, 143 were eventually intubated for acute respiratory failure caused by lung disease. Of these, 84 were excluded, because the patients were already tracheotomized prior to the ICU admission (n = 14), they were transferred from other institutions and only limited data were available (n = 4), or they were intubated without a trial of HFNC/NPPV (n = 66). The remaining 59 patients were included in this study. These patients were divided into 2 groups according to the time until intubation from the start of HFNC/NPPV; ≤ 6 hours failure (n = 26) and > 6 hours failure group (n = 33, Fig 1).

The baseline characteristics of these two groups are summarized in Table 1, including the age, reason for acute respiratory failure, SOFA score using the worst values of the variables within 24 hours, the P_a_O_2_/FiO_2_ ratio before intubation, and the P_a_CO_2_ value before intubation. The median time until intubation from the start of HFNC/NPPV was 1.51 (0.67–3.04) hours in the ≤ 6 hours failure group and 20.0 (11.0–55.9) hours in the > 6 hours failure group (Table 1). Among 59 patients, 22 patients died at ICU discharge: 4 infectious pneumonia, 7 intestinal pneumonia, 5 ARDS, 1 COPD, and 5 others (2 liver cirrhosis, 2 cancer, 1 alveolar hemorrhage).

**Table 1:** Baseline characteristics of the subjects.

Variable	HFNC / NPPV failure		*p value*
	≤ 6 hours failure (N = 26)	> 6 hours failure (N = 33)	
Age, yr	67.5 (55.8–74.5)	68.0 (54.0–73.5)	0.99
Sex, Male, n (%)	13 (50.0)	22 (66.7)	0.29
BMI, kg/m^2^	20.1 (18.4–21.6)	21.6 (17.7–25.6)	0.26
Past medical history			
Lung disease	10 (38.5)	12 (36.4)	> 0.99
Obstructive airway diseases	4 (15.4)	4 (12.1)	0.72
Interstitial lung disease	4 (15.4)	3 (9.1)	0.69
Lung cancer	0 (0.0)	1 (3.0)	> 0.99
Heart disease	6 (23.1)	9 (27.3)	0.77
Hematological cancer	5 (19.2)	7 (21.2)	> 0.99
Dementia	2 (7.7)	1 (3.0)	0.58
Reason for acute respiratory failure			
Infectious lung diseases, n (%)	8 (30.8)	15 (45.5)	0.29
Interstitial lung diseases, n (%)	5 (19.2)	5 (15.2)	0.74
Obstructive airway diseases	1 (3.9)	1 (3.0)	> 0.99
Extrapulmonary ARDS, n (%)	7 (26.9)	6 (18.2)	0.53
Others, n (%)	5 (19.2)	6 (18.2)	> 0.99
SOFA score*	12.0 (8.0–16.0)	9.0 (6.0–15.0)	0.17
Alb, g/dL	2.8 (2.3–3.2)	2.5 (2.2–3.0)	0.14
BUN, mg/dL	29.0 (17.1–79.1)	27.1 (16.5–48.3)	0.72
Cre, mg/dL	0.9 (0.5–2.2)	1.0 (0.7–2.0)	0.84
CRP, mg/dL	8.1 (3.1–19.7)	10.3 (4.5–21.1)	0.34
PaO_2_/FiO_2_ ratio before intubation, mmHg	128.0 ± 54.4	113.6 ± 41.5	0.23
PaCO_2_ before intubation, mmHg	47.5 ± 22.2	43.0 ± 16.9	0.49
Proportion of use of HFNC, n (%)	15 (57.7)	22 (66.7)	0.59
Proportion of use of NPPV, n (%)	14 (53.9)	18 (54.6)	> 0.99
PaO_2_/FiO_2_ ratio before starting devices, mmHg	157.2 ± 111.3	156.0 ± 88.9	0.68
PaCO_2_ before starting devices, mmHg	38.5 ± 14.5	38.4 ± 17.0	0.54
Time to failure from starting devices, hours	1.5 (0.7–3.0)	20.0 (11.0–55.9)	< 0.01
Time to starting devices from admission, hours	0.0 (0.0–4.5)	0.0 (0.0–19.0)	0.32
Time to intubation from admission, hours	3.0 (1.0–6.0)	42.0 (19.5–93.5)	< 0.01

Data are presented as the median and interquartile ranges (25–75% percentile), mean ± standard deviation, or absolute frequencies with percentages.

*Abbreviation*: *BMI*, body mass index; *ARDS*, acute respiratory distress syndrome; *SOFA score*, Sequential Organ Failure Assessment score; *TP*, total protein; *Alb*, albumin; *BUN*, blood urea nitrogen; *Cre*, creatinin; *CRP*, c-reactive protein; *HFNC*, high flow nasal cannula; *NPPV*, noninvasive positive pressure ventilation.

*SOFA score was calculated by using the worst values within 24 hours.

### Analysis of the ICU mortality between the 2 groups

The mortality rates at the time of discharge from the ICU were 23.1% (6/26) and 48.5% (16/33) in the ≤ 6 hours failure and > 6 hours failure groups, respectively. We performed pairwise comparisons with univariate and multivariate logistic regression models (model 1), which showed a statistically significantly higher mortality in the > 6 hours failure group as compared to the ≤ 6 hours failure group: the crude odds ratio (OR) and adjusted OR were 3.1 (1.0–9.8) and 8.8 (1.9–40.6), respectively (Table 2).

**Table 2:** Odds of primary and secondary outcome.

Variable	Univariate		Multivariate (model 1)		Multivariate (model 2)	
	Crude Odds (95%CI)	*p value*	Adjusted Odds (95%CI)	*p value*	Adjusted Odds (95%CI)	*p value*
Primary Outcome						
ICU mortality, > 6 hrs** (vs. ≤ 6 hrs*)	3.14 (1.00–9.80)	0.05	8.82 (1.92–40.56)	< 0.01	24.45 (2.64–226.75)	< 0.01
Secondary Outcome						
30 days mortality, > 6 hrs** (vs. ≤ 6 hrs*)	3.14 (1.00–9.80)	0.05	7.72 (1.78–33.51)	< 0.01	16.02 (2.31–111.31)	0.01
Ventilator weaning ratio, > 6 hrs** (vs. ≤ 6 hrs*)	0.42 (0.15–1.20)	0.11	0.24 (0.07–0.83)	0.02	0.22 (0.06–0.81)	0.02

Multivariate analysis was performed by 3 adjusting factors (model 1; age, SOFA score, and PaCO_2_ before intubation) or 5 factors (model 2; age, reason for acute respiratory failure, SOFA score, and PaCO_2_ and PF ratio before intubation). vs. means the reference value.

*≤ 6 hrs means the group who had the failure of HFNC/NPPV within 6 hours from the initiation.

**> 6 hrs means the group who had the failure of HFNC/NPPV at least 6 hours after the initiation.

As secondary outcomes, the > 6 hours failure group also showed a higher 30-day mortality (7.7 [1.8–33.5]) and decreased proportion of ventilator weaning (0.2 [0.1–0.8]), as compared to the ≤ 6 hours failure group (Table 2). These results were similar even if we used different adjustment factors (model 2). Ratios of tracheostomy at their discharge were similar between the two groups (27% [7/26] in ≤ 6 hours failure groups, and 21% [7/33] in > 6 hours failure groups, *p* = 0.76).

### Analysis of association between ICU mortality and time to failure

**Fig 2:**
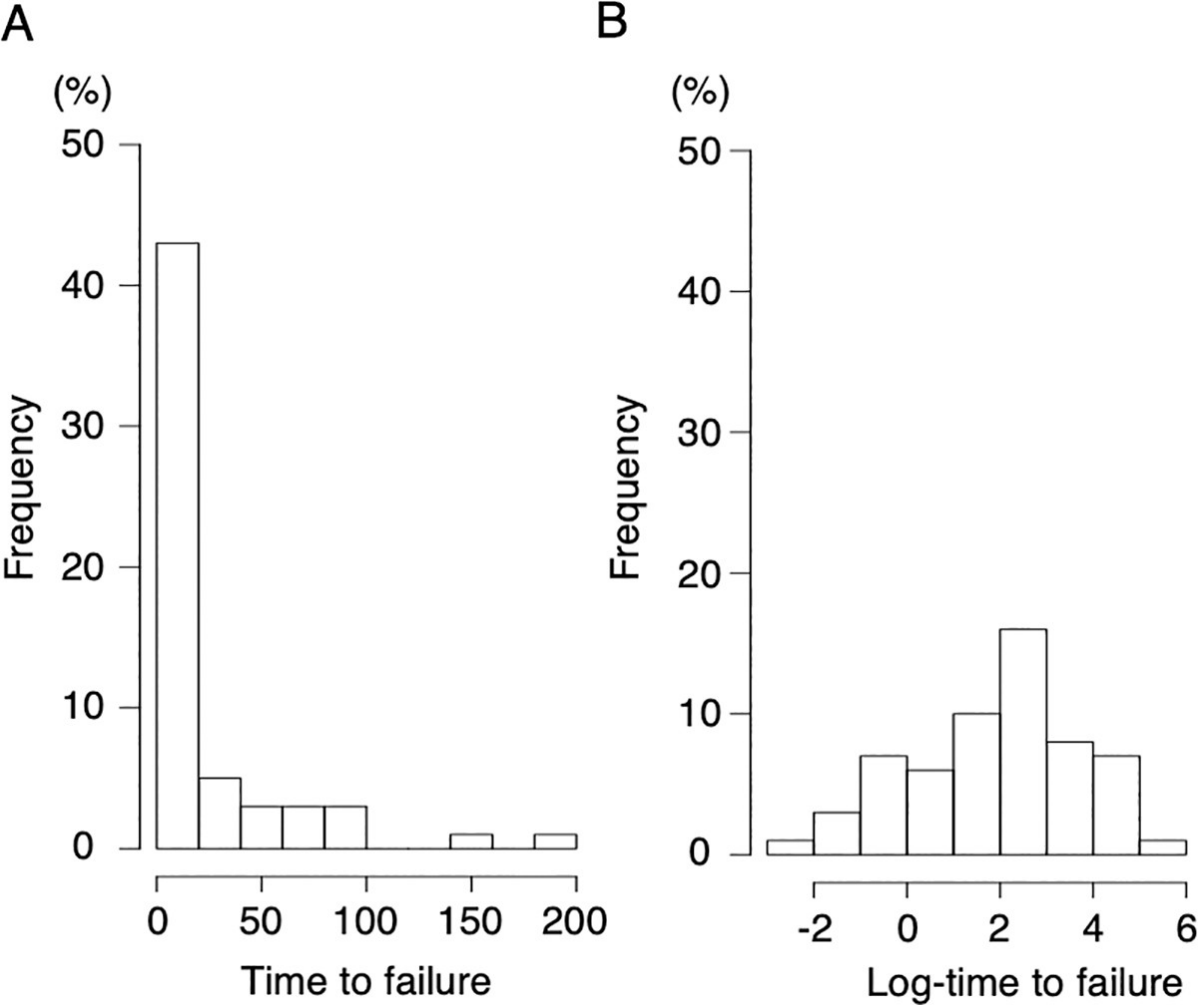
Distribution of time to failure of HFNC/NPPV. Distribution of time to failure of HFNC/NPPV (A) and log-transformed value of time to failure of HFNC/NPPV (B).

**Fig 3:**
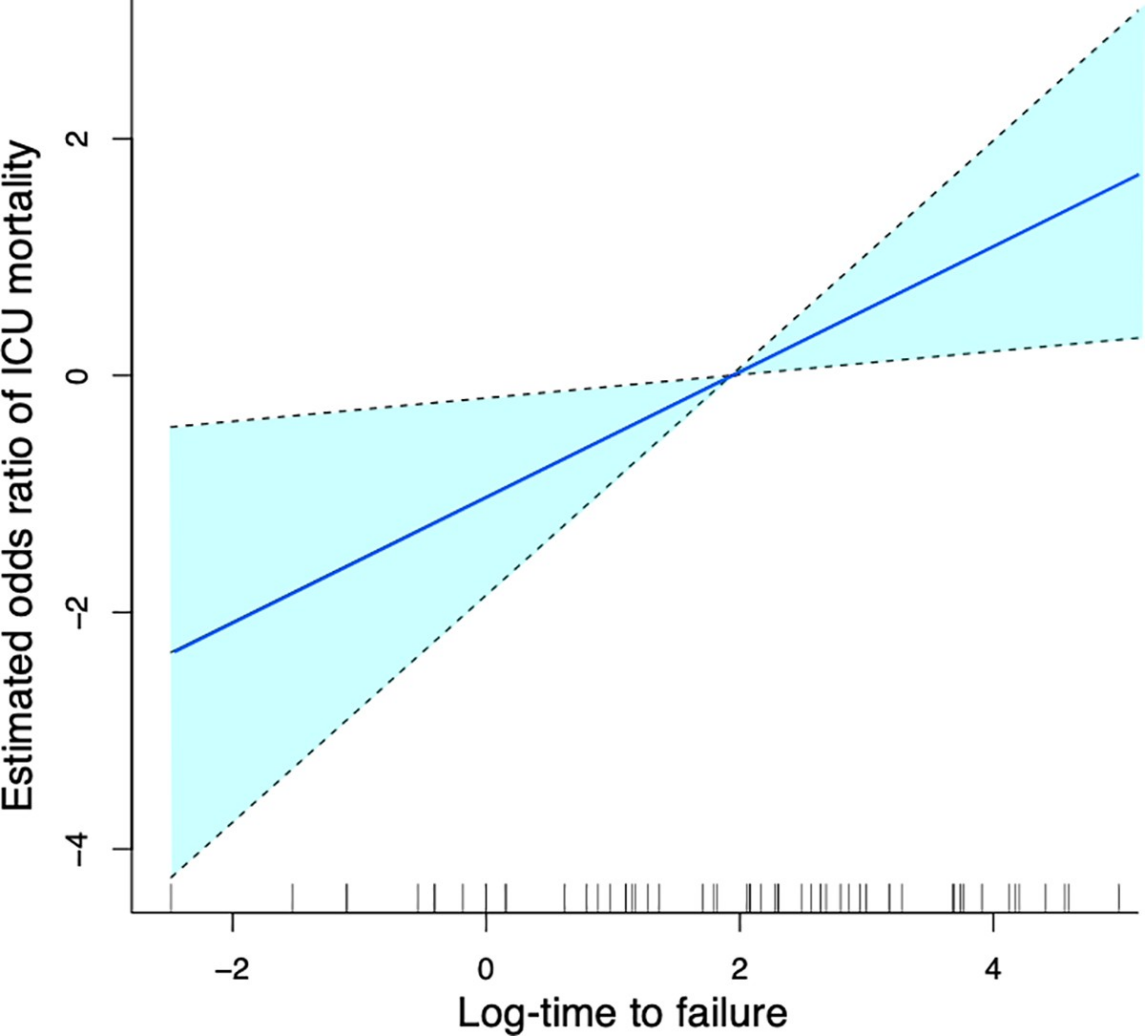
Spline curve in log odds ratio on the effect of log-time to failure on ICU mortality. Spline curve was plotted by using 3 adjusting factors (age, SOFA score, and PaCO2 before intubation). Dotted line means 95% confidence interval.

We also evaluated the relationship between ICU mortality and the time to failure of HFNC/NPPV using another analytical approach without categorizing the time to failure of HFNC/NPPV. With a normalized distribution of the log-transformed time to failure (Fig 2), the fitted spline curve of log odds of ICU mortality rate showed linearity with a positive slope for log-transformed time to failure (Fig 3). Following this result, we performed a multivariable logistic regression analysis by handling the time to failure of HFNC/NPPV as a continuous variable. The results showed that longer durations before failure of HFNC/NPPV was statistically significantly associated with ICU mortality (OR: 1.7 [95%CI: 1.1–2.6], *p* < 0.01) (Table 3). Taken together with the result of the spline curve, this result suggests that longer durations before failure of HFNC/NPPV is associated with a higher probability of ICU mortality.

**Table 3:** Multivariate analysis with handling the time to failure of HFNC/NPPV as a continuous variable.

Variable	OR (95% CI)	p value
Age	0.98 (0.93–1.03)	0.37
SOFA score	1.31 (1.09–1.59)	< 0.01
PaCO2 before intubation	1.05 (1.01–1.08)	0.01
Log-transformed time to failure of NHFC/NPPV	1.70 (1.11–2.59)	< 0.01

OR = Odds ratio; 95% CI = 95% confidence interval.

### Survival analysis

**Fig 4:**
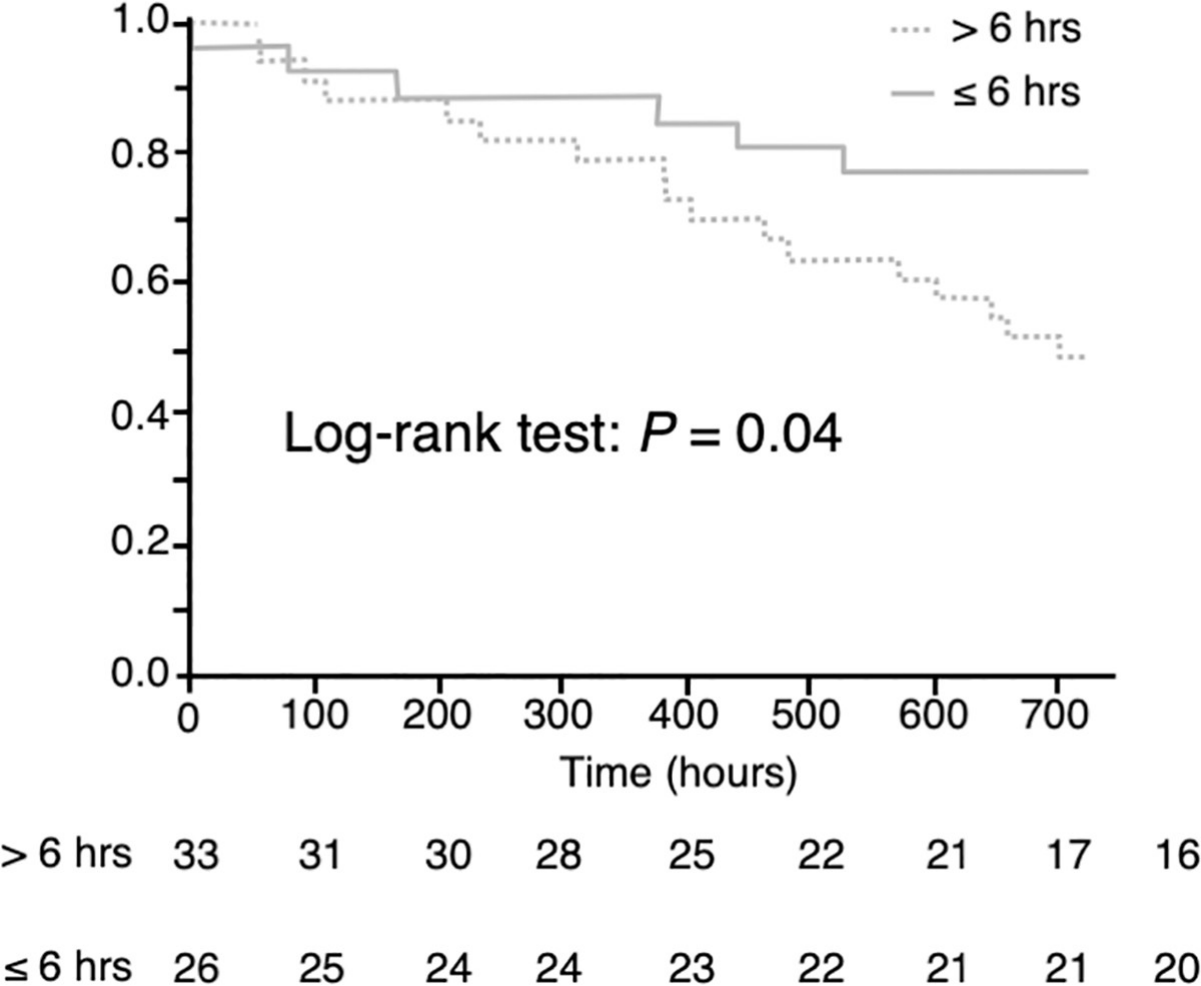
Survival analysis. The cumulative survival rates in the group with the failure of HFNC/NPPV > 6 hours after it was attempted (> 6 hours, dotted line), as compared to those in the group with the failure of HFNC/NPPV ≤ 6 hours after it was attempted (≤ 6 hours, gray line) groups. The p value was calculated using the log-rank test.

The cumulative survival rate in the > 6 hours failure group was compared with those in the ≤ 6 hours failure groups by the Kaplan-Meier method. The log-rank test revealed a statistically significant difference between the two groups (*p* = 0.04, Fig 4).

## Discussion

A previous study showed that delayed intubation following attempted use of HFNC was associated with an increased mortality rate in patients with acute respiratory failure [15]. However, the precise definition of “delay” remains unclear. Our retrospective study demonstrates that intubation for failure of HFNC/NPPV even as early as 6 hours after the attempted use of these non-invasive ventilatory support methods was associated with increased ICU mortality in patients with acute respiratory failure caused by lung disease. To the best of our knowledge, this is is the first study to show that failure of non-invasive respiratory support at less than 48 hours after the institution of such support was associated with increased ICU mortality.

We set the time of 6 hours after the start of non-invasive respiratory support as the cutoff time to dichotomize the patients for analysis. According to several guidelines, if we treat the patients with non-invasive respiratory support, the evaluation every 4–6 hours is recommended to judge whether we should continue this support or switch to treatment using mechanical ventilation because a positive response to treatment should certainly be evident at 4–6 hours after start of the support [16, 17]. Actually, as with our study, many previous prospective and retrospective studies have used this time cutoff point to evaluate the effect of non-invasive respiratory support [18–20]. Based on this evidence, we believe that the time of 6 hours is one of the most suitable time windows for evaluating the effect of non-invasive respiratory support and considering the need for intubation. Our analysis of spline curve suggests another possibility that earlier cutoff point time for the failure such as 1 hour could still be associated with worse outcomes, but we were unable to evaluate this due to the small size who had failure in even earlier than our 6 hours time window (only 11 were the patients with the failure within 1 hour in our data). Future larger studies are needed for investigation.

The mechanism for the association between failure of non-invasive respiratory support and worse outcome has not been completely understood, but some previous studies have discussed a potential relationship with delayed intubation caused by attempting NPPV/HFNC [12, 25, 26]. Given the limited physiologic reserve for patients with acute respiratory failure, it is not surprising to consider that longer exposure of insufficient oxygenation and hypercapnia caused by delayed intubation should be avoided as much as possible. In fact, our data suggests that the > 6 hours failure group had a longer duration from the time of admission until intubation compared with the ≤ 6 hours failure group (3 hours vs 42 hours) and this difference might have contributed to worse outcomes in this group.

Some previous studies have showed that failure of non-invasive respiratory support was associated with an increase in the risk of intubation-related complications [12] and higher expired tidal volumes, which is a known risk for pulmonary barotrauma [27], both which may have also contributed to increased mortality in ICU patients although further investigation would be needed.

Our study does not suggest that non-invasive respiratory support should not been attempted at all; rather, we propose the importance of correct, early judgment for the transition to mechanical ventilation in patients who have received HFNC/NPPV but may likely fail. While many previous studies have shown the beneficial effect of NPPV/HFNC for many kinds of patients, there are certain populations who are nonresponsive to this support. Based on our data, it may be important not to overlook any clinical signs for failure within 6 hours and intubate the patients who would fail without hesitation, before the patient crashes. It is challenging to determine the patients who would fail in advance, but interestingly, new indicators and scoring systems have been developed for predicting HFNC/NPPV failure more precisely [28, 29]. These tools enable us to judge earlier and more precisely as to the need for intubation, which may help contribute to improved outcomes.

The higher mortality rate in the > 6 hours failure group as compared to that in the ≤ 6 hours failure group may not be attributable to the harmful effects of failure of HFNC/NPPV as discussed above but may be simply due to the differences in the pathophysiology between the > 6 hours failure group and ≤ 6 hours failure group. For example, acute deterioration may be evidence that a patient’s clinical course is benign because it can also be acutely responsive to treatments. The acute respiratory failure in some patients of the ≤ 6 hours failure group may have substantial contribution of other, non-pulmonary concerns, such as cardiogenic failure, that may be amenable to appropriate treatment; however, patients in the > 6 hours failure group usually show a less dynamic clinical course and this subacute or chronic time course until intubation may mean it may take a longer time to recover. If we can identify the patients who have more chronic clinical course and likely fail non-invasive respiratory support, it may be better to intubate them without any attempt of non-invasive respiratory support or at least in shortest time possible after initial attempt in order to avoid delayed intubation. We should balance between the risks of delayed intubation and benefits for non-invasive respiratory support.

Our study may be unique because both patients receiving HFNC and NPPV were included in the analysis. Although NPPV and HFNC are similar in that they both improve oxygenation without the need for recourse to invasive mechanical ventilatory support [30, 31], some functions obviously differ. Generally speaking, NPPV has the advantage of also reducing CO_2_ accumulation in addition to improving oxygen saturation, because it allows for tidal volume to be increased under the setting of pressure support. On the other hand, HFNC has the advantage of greater comfort associated with the fitting of the device. However, in actual clinical practice, this theory may not be applicable to every patient in every situation, and sometimes it is difficult to decide which device would be better for the patient until we actually try both devices. Actually, in our data, both NPPV and HFNC were attempted at least one time before intubation for 17% (10/59) of the patients who failed, and even if we incorporate the variable of the choice of NPPV or HFNC into multivariate logistic regression analysis, statistically significant association was still observed (data was not shown). Considering that it is often possible to switch between HFNC and NPPV without difficulty [32, 33], we believe that the results of our study are worthwhile, because they reflect the outcomes under actual use conditions of these devices.

There were some limitations of this study. First, it was a small-sized, single-center, retrospective study, and it may therefore lack generalizability. Second, we do not have any clinical information before patients began non-invasive respiratory support, such as P/F ratio at their hospital admission before intubation. Finally, we did not analyze the causes for intubation in the patients with HFNC/NPPV failure. Investigations in the future of the association between the causes of intubation and mortality would be useful for understanding the reason(s) underlying the increased mortality in patients with HFNC/NPPV failure.

## Conclusions

The ICU mortality in patients with acute respiratory failure caused by lung diseases was increased if the time until failure of HFNC and NPPV was more than 6 hours.
